# Supplementary material for: Wafer-scale functional circuits based on two dimensional semiconductors with fabrication optimized by machine learning
Source: Nat Commun. 2021 Oct 12;12:5953. doi: 10.1038/s41467-021-26230-x (PMC8511068; doi:10.1038/s41467-021-26230-x)
Supplement: Supplementary file 1 — Supplementary Information [file 41467_2021_26230_MOESM1_ESM.docx]

**Supplementary Information**

**Wafer-Scale Functional Circuits Based on Two Dimensional Semiconductors with Fabrication Optimized by Machine Learning**

Xinyu Chen^†1^, Yaochen Sheng^†1^, Yufeng Xie^†1^, Hongwei Tang^†1^, Zeming Wang^1^, Yu Wang^1^, Yin Wang^1^, Fuyou Liao^1^, Jingyi Ma^1^, Xiaojiao Guo^1^, Ling Tong^1^, Hanqi Liu^1^, Hao Liu^1^, Tianxiang Wu^1^, Jiaxin Cao^1^, Sitong Bu^1^, Hui Shen^1^, Fuyu Bai^1^, Daming Huang^1^, Jianan Deng^2^, Antoine Riaud^1^, Zihan Xu^3^, Chenjian Wu^4^, Shiwei Xing^4^, Ye Lu^2^, Shunli Ma^1^, Zhengzong Sun^1^, Zhongyin Xue^5^, Zengfeng Di^5^, Xiao Gong^6^, David Wei Zhang^1^, Peng Zhou^*1^, Jing Wan^*2^, and Wenzhong Bao^*1^

^1^ State Key Laboratory of ASIC and System, School of Microelectronics, Fudan University, Shanghai 200433, P. R. China.

^2^ State Key Laboratory of ASIC and System, School of Information Science and Technology, Fudan University, Shanghai 200433, P. R. China.

^3^ Shenzhen Six Carbon Technology, Shenzhen 518055, P. R. China.

^4^ School of Electronic and Information Engineering, Soochow University, Suzhou 215006, P. R. China.

^5^ State Key Laboratory of Functional Materials for Informatics, Shanghai Institute of Microsystem and Information Technology, Chinese Academy of Sciences, 865 Changning Road, Shanghai, 200050, China

^6^ Department of Electrical and Computer Engineering, National University of Singapore, Singapore 117583, Singapore.

*Correspondence to pengzhou@fudan.edu.cn; jingwan@fudan.edu.cn; baowz@fudan.edu.cn.

† These authors contribute to this work equally.

Table of Contents

[**Supplementary Notes (including Figures and Tables)** 3](#_Toc81680488)

[**1 - Raman spectra and mapping of wafer-scale MoS_2_ continuous films** 3](#_Toc81680489)

[**2 - Detailed processing steps for fabricating MoS_2_ TG-FETs** 4](#_Toc81680490)

[**3 -** **The supervised ML method used in this work** 6](#_Toc81680491)

[**4 - Impact of contact electrode on the transfer characteristics for MoS_2_ TG-FETs** 7](#_Toc81680492)

[**5 - Impact of seeding layer on the transfer characteristics for MoS_2_ TG-FETs** 9](#_Toc81680493)

[**6 - Transfer characteristics of MoS_2_ TG-FETs with and without annealing after seeding layer deposition** 10](#_Toc81680494)

[**7 - Impact of TG electrodes on the transfer characteristics for MoS_2_ TG-FETs** 11](#_Toc81680495)

[**8 - Impact of seeding layer on the hysteresis characteristics for MoS_2_ TG-FETs** 13](#_Toc81680496)

[**9 - Comparison of an overall performance for MoS_2_ TG-FETs** 14](#_Toc81680497)

[**10 - Low-frequency 1/f noise in top-gated MoS_2_ FETs** 16](#_Toc81680498)

[**11 - Analysis of inverters with different pull-up transistors** 18](#_Toc81680499)

[**12 - Analog amplifier composed of an inverter based on MoS_2_ FET** 19](#_Toc81680500)

[**13 - Measurement results of various basic logic circuit units** 20](#_Toc81680501)

[**14 - The limiting factor for high frequency operation of MoS_2_ integrated circuits** 21](#_Toc81680502)

[**15 - Schematic diagram of DRAM testing setup** 22](#_Toc81680503)

[**16 - Photoelectric characteristics for top-gated MoS_2_ FETs** 23](#_Toc81680504)

[**17 -** **Discussion of the origins for failed circuits in Fig. 5** 25](#_Toc81680505)

[**18 - Comparison of our work with recently published results** 26](#_Toc81680506)

[**Supplementary References** 27](#_Toc81680507)

**Supplementary Note 1 - Raman spectra and mapping of wafer-scale MoS_2_ continuous films**

Raman spectra mapping from 1800 points (insets in Supplementary Figure 1a-c) were performed in 3 separate regions (a, b, and c in Supplementary Figure 1d) of the as-synthesized MoS_2_ film. The Raman spectra results have nearly identical shapes and intensities, revealing large-area uniformity in our monolayer MoS_2_ film. Raman mapping at the $E_{2g}^{1}$ peak (384.4 cm^−1^) was also performed to build a 2D color plot in a square region (120×120 μm^2^) at these regions over the wafer. These results show that the synthesis method used in this work can effectively prepare wafer-level, highly crystalline and uniform MoS_2_ monolayers.


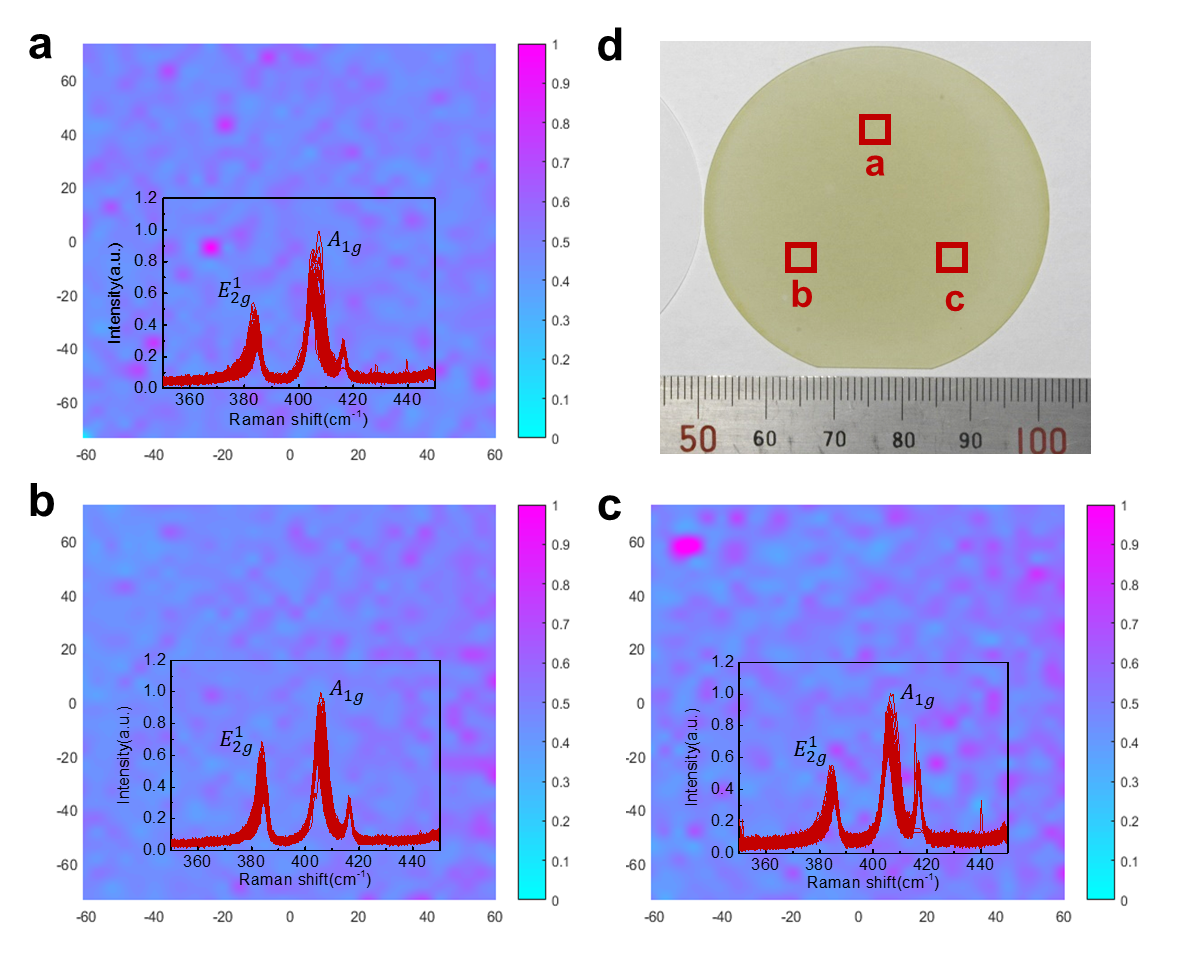


**Supplementary Figure 1.** Raman intensity maps normalized by the highest intensity at the $E_{2g}^{1}$ peak (384.4 cm^−1^) in wafer-scale monolayer MoS_2_ grown on a sapphire substrate, where the scanning area is 120×120 μm^2^. The wavelength of the laser source is 514 nm. The insets in **a-c** show normalized Raman spectra from 1800 different locations in the scan area. **d,** Optical images of wafer-scale monolayer MoS_2_, in which the three red squares (a, b, and c) indicate the scanning areas in **a-c**, respectively.

**Supplementary Note 2 - Detailed processing steps for fabricating MoS_2_ TG-FETs**

Supplementary Table 1 shows detailed MoS_2_ FET processing steps used for machine learning in Fig. 2c in the main text. Steps filled with color are marked in Fig. 2c in the main text, where device performance (*V*_T_ or mobility *μ*) is more sensitive to changes in these parameters. In the following Supplementary Notes 4–9, we discuss the optimization of these steps.

**Supplementary Table 1.** Detailed processing steps for fabricating MoS_2_ TG-FETs.

| Step Number | Detailed Process | Step Number | Detailed Process |
| --- | --- | --- | --- |
| 1 | Quality of MoS_2_ film | 26 | Deposition rate 2 of contact |
| 2 | 1^st^ Photoresist coating | 27 | 3^rd^ Peeling temperature |
| 3 | 1^st^ Baking temperature | 28 | 3^rd^ Peeling time |
| 4 | 1^st^ Baking time | 29 | Annealing machine of contact |
| 5 | 1^st^ Spinning speed | 30 | Annealing gas of contact |
| 6 | 2^nd^ Photoresist coating | 31 | Annealing temperature of contact |
| 7 | 2^nd^ Baking temperature | 32 | Annealing time of contact |
| 8 | 2^nd^ Baking time | 33 | Annealing machine of seeding layer |
| 9 | 2^nd^ Spinning speed | 34 | Annealing time of seeding layer |
| 10 | 2^nd^ Exposure dose | 35 | Annealing temperature of seeding layer |
| 11 | 2^nd^ Developing time | 36 | Annealing gas of seeding layer |
| 12 | Etching gas | 37 | Seeding layer |
| 13 | Etching time | 38 | Temperature of the main dielectric growth |
| 14 | Etching power | 39 | Thickness of the main dielectric |
| 15 | Vacuum level of etching | 40 | Materials of top gate |
| 16 | 2^nd^ Peeling temperature | 41 | Deposition method of top gate |
| 17 | 2^nd^ Peeling time | 42 | Vacuum level of top gate deposition |
| 18 | 3^rd^ Photoresist coating | 43 | Deposition rate 1 of top gate |
| 19 | 3^rd^ Baking temperature | 44 | Deposition rate 2 of top gate |
| 20 | 3^rd^ Baking time | 45 | 4^th^ Photoresist coating |
| 21 | 3^rd^ Spinning speed | 46 | 4^th^ Baking temperature |
| 22 | Deposition method of contact | 47 | 4^th^ Baking time |
| 23 | Vacuum level of contact deposition | 48 | 4^th^ Spinning speed |
| 24 | Contact materials | 49 | 4^th^ Peeling temperature |
| 25 | Deposition rate 1 of contact | 50 | 4^th^ Peeling time |

**Supplementary Note 3 - The supervised ML method used in this work**

a. Description of ensemble learning:

Because the semiconductor manufacturing process cannot guarantee similar data types in each step, we choose the Random-Under-Sampling (RUSBoost) algorithm and the decision tree as the weak classifier that can efficiently handle discrete data, and RUSBoost is especially effective at classifying imbalanced data.

The RUS algorithm takes N, the number of members in the class with the fewest members in the training data, as the basic unit for sampling. Classes with more members are sampled by taking only N observations of every class. After training the RUSBoost classifier, we can find the best semiconductor manufacturing recipe combination through a grid search method. Grid search is extensively used in the field of discrete parameters.

b. Description of Radom forest algorithm

Random forest is an integrated learning algorithm based on decision tree learners, which is widely used and easy to implement^1,2^. The random forest algorithm can be summarized as follows:

1) The bootstrap method is used to select *n* samples from the sample set as a training set.

2) A decision tree is generated from the sample set. Multiple features *X_j_* are randomly selected at each generated node *m* without repetition. Then they are used to divide the sample set until finding the best division feature.

3) Step 1 and step 2 are repeated *k* times, where *k* is the number of decision trees in the random forest.

4) The trained random forest is used to predict the test sample, while the voting method is used to determine the prediction result.

c. Feature importance assessment

Importance evaluation is used to see how much each feature contributes to each tree in the random forest. The Gini index was used for evaluation, which is defined as follows:

$Gini\left( p \right)=\sum_{k=1}^{k} p_{k}\left( 1-p_{k} \right)=1-\sum_{k=1}^{k} p_{k}^{2}$ (1)

where *k* is the number of categories and $p_{k}$ is the weight of category *k*.

For feature *X_j_* at node *m* in the Gini index, the difference before and after the branch generation of node *m* is defined as:

${VIM}_{jm}^{(Gini)}={GI}_{m}-{GI}_{l}-{GI}_{r}$ (2)

where *GI_l_* and *GI_r_* represent the Gini indices of two new nodes after the branch, respectively.

For feature *X_j_* that appears in decision tree *i*, if the node is in set *M*, then the importance of *X_j_* in decision tree *i* is:

${VIM}_{ij}^{(Gini)}=\sum_{m\in M} {VIM}_{jm}^{(Gini)}$ (3)

If there are *n* trees in the random forest, then

${VIM}_{j}^{(Gini)}=\sum_{i=1}^{n} {VIM}_{ij}^{(Gini)}$ (4)

Finally, all the obtained importance scores are normalized:

${VIM}_{j}=\frac{{VIM}_{j}}{\sum_{i=1}^{c} {VIM}_{i}}$ (5)

where the denominator is the sum of all features’ importance scores, and the numerator is the Gini index of feature *j*.

d. How to determine evaluation score

In this work, the overall evaluation index of the MoS_2_ FETs is contributed by the scores of mobility (*μ*), threshold voltage (*V*_T_), subthreshold swing (*SS*) and current on-off ratio (*I*_on_/*I*_off_) with weights of 30%, 30%, 20% and 20%, respectively. The percentage values can be adjusted according to the specific requirement of FETs, e.g., high mobility is more important for faster operation speed and *SS* for low power consumption. Then the random forest algorithm is used to predict the scores of all possible processing combinations.

The random forest algorithm can classify the sample set composed of different process combinations and device performance by using a weak classifier that can effectively process discrete data and evaluate the importance of each process step, which is mainly obtained through the Gini index evaluation. According to the evaluation result, several process steps such as the material quality, contact deposition, seeding layer, and TG deposition display the most obvious influence on the final performance of the device. All of them can be related to their corresponding physical mechanisms such as defects of MoS_2_, Schottky barrier, interfacial scattering, work function and interface charge traps of TG metal. However, the detailed physical explanations are not the focus of this work. Then a score predictor based on the random forest algorithm was adopted to predict the results from all possible process combinations obtained by a grid search method, followed by realistic experiments which provide feedback and further improve the reliability of the score predictor.

**Supplementary Note 4 - Impact of contact electrode on the transfer characteristics for MoS_2_ TG-FETs**

For the contact between MoS_2_ and the metal electrode, two aspects affect the contact resistance: 1) the tunneling barrier between the metal and MoS_2_ below the metal contact because of the van der Waals (vdW) gap and, 2) the Schottky barrier between the MoS_2_ channel and the metal electrodes. In order to compare the influence of different metal contacts on device electrical performance, four types of electrodes (Ti/Au, Au, In/Au and Ag/Au) were deposited as source-drain electrodes (Supplementary Figure 2). The results show that in this comparison group, the devices with Au or Ti/Au electrodes exhibit larger on-state current, which is indicative of a better contact. The fabrication recipes are shown in Supplementary Table 2.

**Supplementary Table 2.** Comparison group in which the S/D contact material is a variable and other parameters are kept the same.

| Process | S/D | Seeding layer | Anneal of SL | Material of TG |
| --- | --- | --- | --- | --- |
| a | Ti/Au | 2 nm SiO_2_ | w/o | Au |
| b | Au | 2 nm SiO_2_ | w/o | Au |
| c | In/Au | 2 nm SiO_2_ | w/o | Au |
| d | Ag/Au | 2 nm SiO_2_ | w/o | Au |


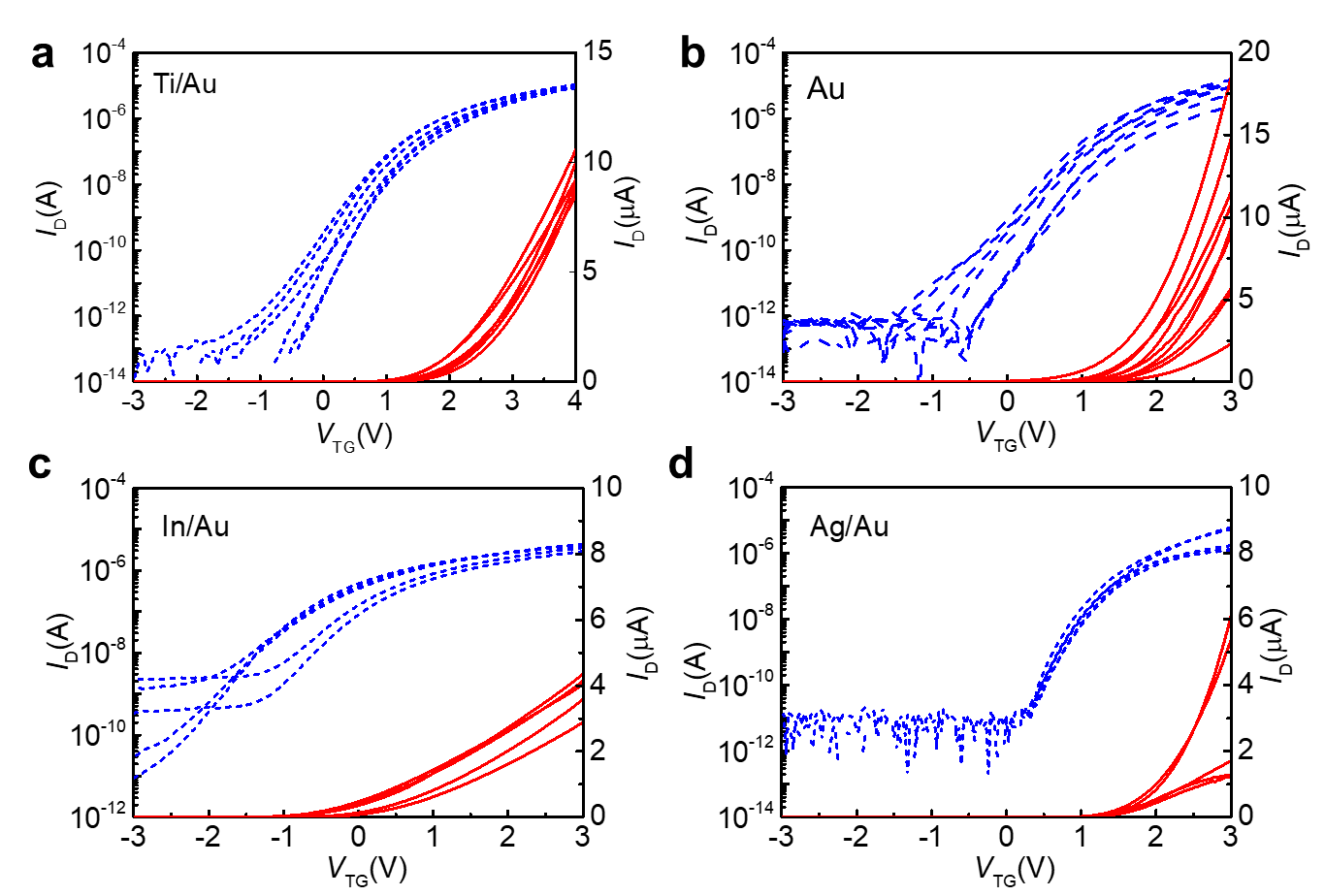


**Supplementary Figure 2.** *I*_D_-*V*_TG_ curves of MoS_2_ TG-FETs with different contacts. *I*_D_-*V*_TG_ curves of MoS_2_ TG-FETs with **(a)** Ti/Au, **(b)** Au, **(c)** In/Au, and **(d)** Ag/Au contacts at *V*_DS_ = 0.1 V. Blue and red curves correspond to the logarithmic scale on the left and linear scale on the right y-axis, respectively.

In this group of comparison experiments, we further compare the performance of top-gated MoS_2_ FETs with different contact electrodes. The contact resistant *R*c extracted using the Y-function method is plotted with error bars for different contact electrodes shown in Supplementary Figure 3. Based on these statistical results, we can again conclude that Au and Ti/Au contact electrodes provide lower contact resistance than those from other electrodes.


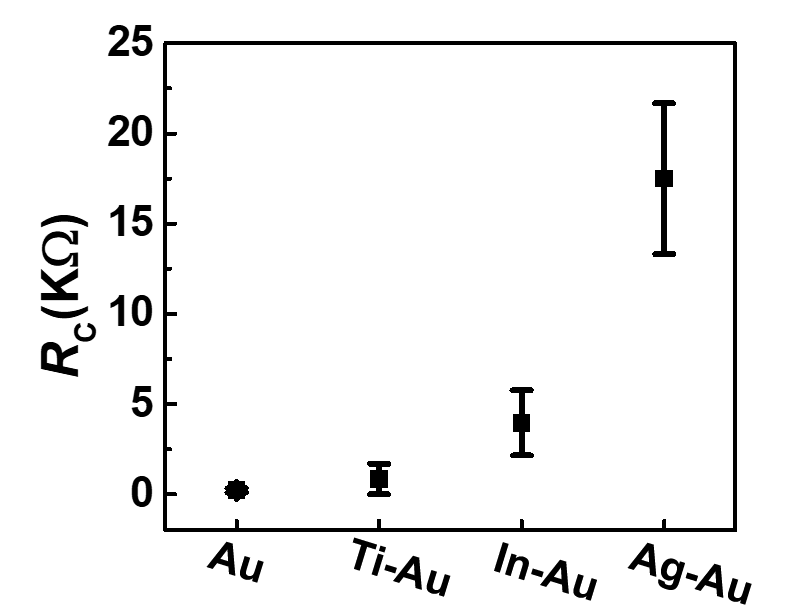


**Supplementary Figure 3.** Extracted contact resistance between MoS_2_ and different metals using the Y-function fitting method. The error bars indicate standard error.

**Supplementary Note 5** **- Impact of seeding layer on the transfer characteristics for MoS_2_ TG-FETs**

In this group of comparison experiments, we tested different materials for seeding layers (SLs), and found that both 2-nm-thick Al_2_O_3_ is the best SL materials for FETs to achieve positive V_T_ and sizeable on-state current. Its device uniformity is also better than other results. The fabrication recipes and the corresponding transfer characteristics for are shown in Supplementary Table 3 and Supplementary Figure 4.

**Supplementary Table 3.** Comparison group in which the SL material is a variable and other parameters are kept the same.

| Process | S/D | Seeding layer | Anneal of SL | Material of TG |
| --- | --- | --- | --- | --- |
| a | Au | N/A | w/o | Au |
| b | Au | 2 nm Y_2_O_3_ | w/o | Au |
| c | Au | 2 nm Al_2_O_3_ | w/o | Au |
| d | Au | 2 nm SiO_2_ | w/o | Au |


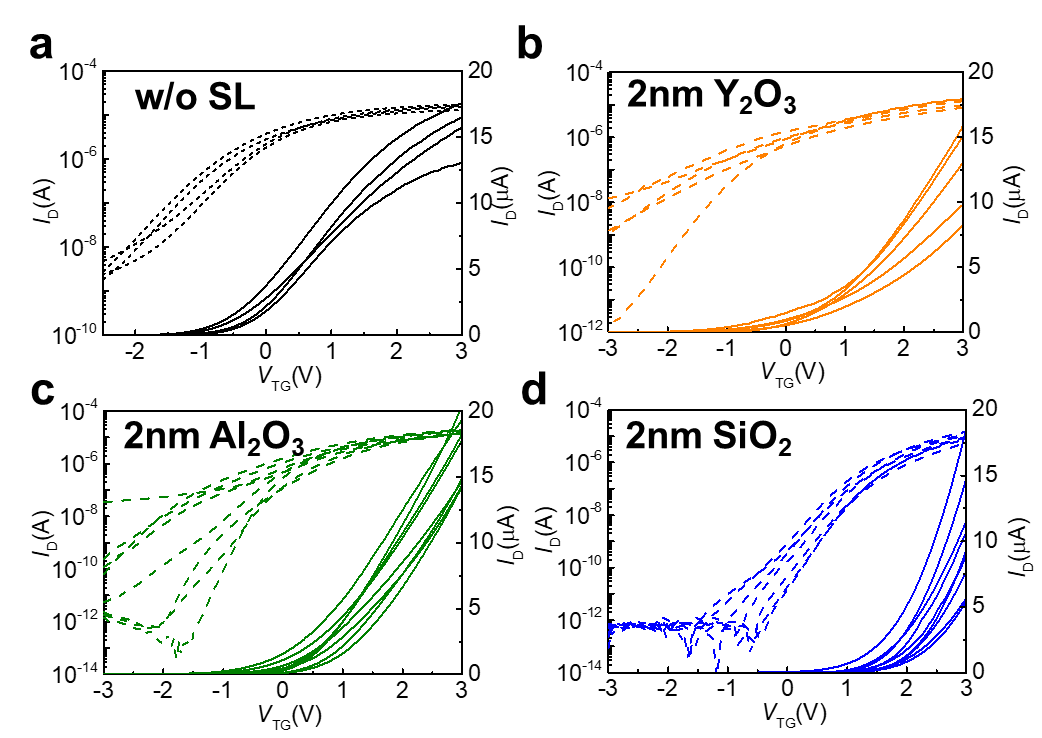


**Supplementary Figure 4.** *I*_D_-*V*_TG_ curves of MoS_2_ TG-FETs with different seeding layers. **a-d,** *I*_D_-*V*_TG_ curves of MoS_2_ TG-FETs with different processing recipes (a-d in Supplementary Table 3) at *V*_DS_ = 0.1 V.

**Supplementary Note 6 - Transfer characteristics** **of MoS_2_ TG-FETs with and without annealing after seeding layer deposition**

In order to improve the electrical characteristics of MoS_2_ FETs, we annealed the devices at 100 °C in nitrogen for 30 min after deposition of a seed layer. Compared with unannealed devices, *V*_T_ is more positive and a steeper subthreshold (SS) is obtained after annealing, most likely due to the improvement of interface between MoS_2_ and seed layer, as well as reduction of defects and dipoles in the channel and contact region. The fabrication recipes and corresponding transfer characteristics are shown in Supplementary Table 4 and Supplementary Figure 5.

**Supplementary Table 4.** Comparison group in which the SL annealing is a variable and other parameters are kept the same.

| Recipe | S/D | Seeding layer | Anneal of SL | Material of TG |
| --- | --- | --- | --- | --- |
| a | Au | 2 nm SiO_2_ | w/o | Au |
| b | Au | 2 nm SiO_2_ | w/ | Au |


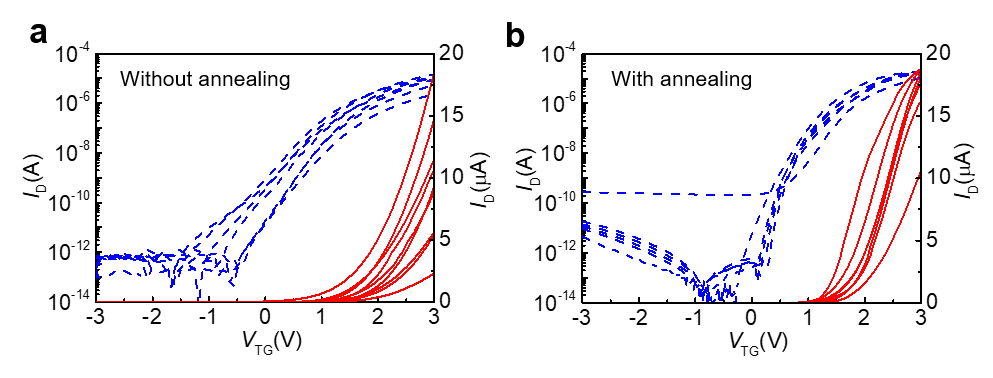


**Supplementary Figure 5.** *I*_D_-*V*_TG_ curves of the MoS_2_ TG-FETs with unannealed and annealed seeding layer. *I*_D_-*V*_TG_ curves of the MoS_2_ TG-FETs on a wafer-scale substrate with (**a)** an unannealed and (**b)** annealed seeding layer at *V*_DS_ = 0.1 V.

**Supplementary Note 7 - Impact of TG electrodes on the transfer characteristics for MoS_2_ TG-FETs**

Au and Al were deposited to fabricate the MoS_2_ TG-FETs. The work function of these metals is used to tune the *V*_T_ of the devices. The experimental results indicate that an Au TG is more desirable for fabricating enhanced-mode FETs due to its larger work function. The fabrication recipes and corresponding transfer characteristics are shown in Supplementary Table 5 and Supplementary Figure 6.

**Supplementary Table 5.** Comparison group in which the TG material is a variable here and other parameters are kept the same.

| Recipe | S/D | Seeding layer | Anneal of SL | Material of TG |
| --- | --- | --- | --- | --- |
| a | Au | 2 nm SiO_2_ | w/ | Au |
| b | Au | 2 nm SiO_2_ | w/ | Al |


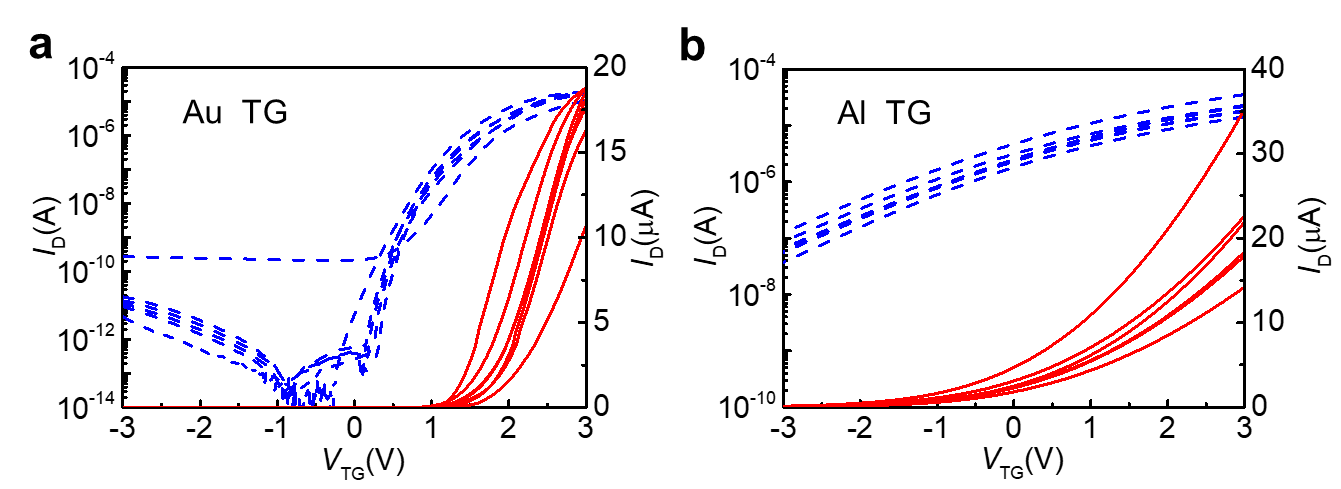


**Supplementary Figure 6.** *I*_D_-*V*_TG_ curves of MoS_2_ TG-FETs with different top gates. *I*_D_-*V*_TG_ curves of MoS_2_ TG-FETs with (**a)** Au TG and (**b)** Al TG at *V*_DS_ = 0.1 V.

Based on the above results, we can tell that the gate metals with different work functions can control the *V*_T_ of the FETs with little effect on the mobility, and the band diagrams of FETs with gate metal work function either greater or smaller than the MoS_2_ work function are shown in Supplementary Figure 7^3^. At zero gate bias, the gate metal with a low work function tends to attract more electrons in the channel, tuning the channel to the charge accumulation regime, while a high work function metal does the opposite. For the Al- and Au-gated MOS_2_ FETs, the *V*_T_ shift is about 2 V and shifts its *V*_T_ from negative to positive. Thus it is beneficial to develop a direct-coupled FET logic (DCFL)^3,4^. Moreover, in this *V*_T_ engineering method, mobility is not influenced because the interface quality between the channel and the dielectric layer is maintained, i.e., no additional carrier scattering is introduced.


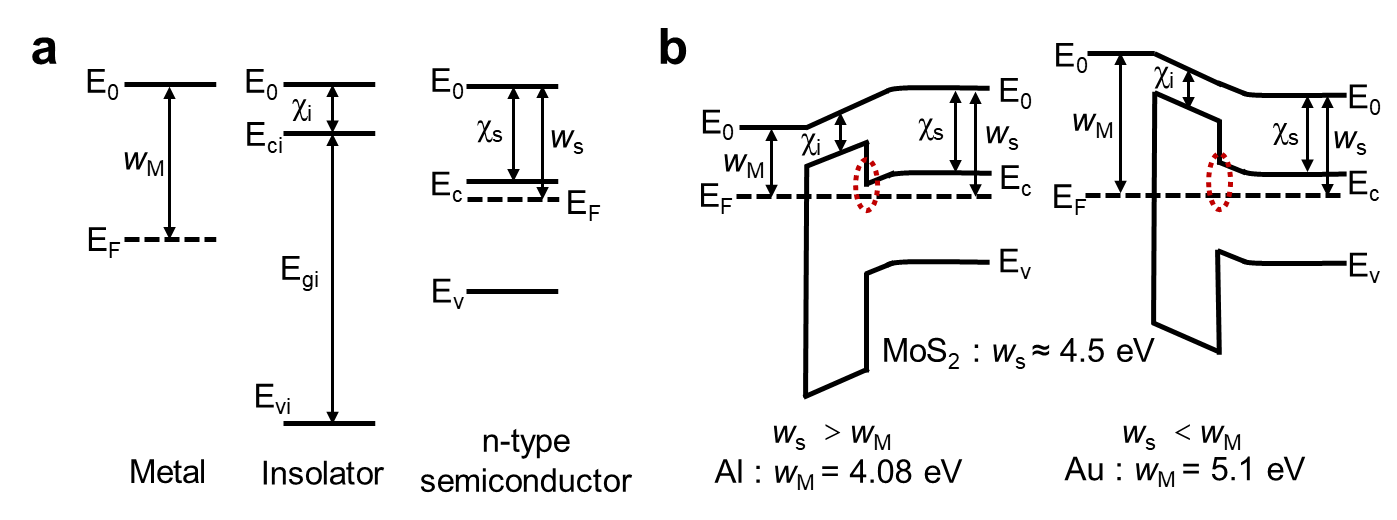


**Supplementary Figure 7.** Energy band analysis for different top gates. Energy band diagrams for **(a)** isolated metal, insulator and semiconductor, and **(b)** after their intimate contact and thermal equilibrium is established^3^.

**Supplementary Note 8 - Impact of seeding layer on the hysteresis characteristics for MoS_2_ TG-FETs**

In this group of comparison experiments, the hysteresis characteristics for MoS_2_ TG-FETs prepared with different seeding layers (SLs) are investigated. The fabrication recipes and dual-sweep transfer characteristics for MoS_2_ TG-FETs are shown in Supplementary Table 6 and Supplementary Figure 8. It is found that the process using 2-nm-thick SiO_2_ as seeding layer has the smallest hysteresis, which indicates that the border trap density of the SiO_2_/HfO_2_ interface is the least^5^. The measurements of transfer curves were all carried out at a gate sweep rate of 100 mV/s (0.03 V resolution), *V*_TG_ from -3 V to 3 V, and hold time of 5s at the beginning and ending points, *V*_DS_ of 0.1 V, and under room temperature and atmospheric pressure. The hysteresis voltage is determined by the *V*_T_ difference between the dual-sweep transfer characteristic curves.

**Supplementary Table 6.** Comparison group in which the SL material is a variable and other parameters are kept the same.

| Process | S/D | Seeding layer | Anneal of SL | Material of TG |
| --- | --- | --- | --- | --- |
| a | Au | N/A | w/o | Au |
| b | Au | 2 nm Y_2_O_3_ | w/o | Au |
| c | Au | 2 nm Al_2_O_3_ | w/o | Au |
| d | Au | 2 nm SiO_2_ | w/o | Au |


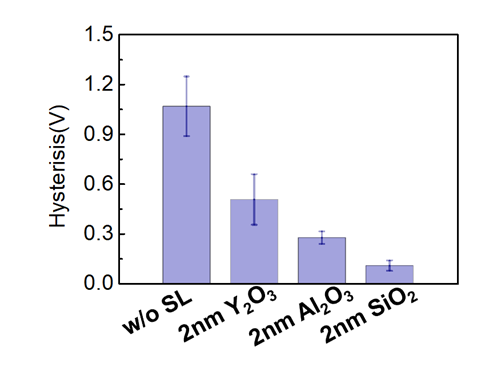


**Supplementary Figure 8.** The hysteresis characteristics of MoS_2_ TG-FETs with different processing recipes (a-d in Supplementary Table 6) at *V*_DS_ = 0.1 V. The error bars indicate standard error.

**Supplementary Note 9 - Comparison of an overall performance for MoS_2_ TG-FETs**

To achieve a wafer-scale homogeneity of device performance, several MoS_2_ TG-FETs arrays were fabricated with different fabrication procedure combinations. Supplementary Figure 9a-c shows more than 100 transfer curves from device arrays prepared through three different process combinations (*a*, *b*, and *c* in Supplementary Table 7). Supplementary Figure 9c is the same as Fig. 2g in the main text and presented here for a straightforward comparison. Other than the four processing steps shown in Supplementary Table 7, all other steps are kept the same for comparison.

**Supplementary Table 7.** Comparison group with 3 different recipe combinations.

| Process  combination | S/D | Seed layer | Anneal of SL | Material of TG |
| --- | --- | --- | --- | --- |

| *a* | Au | 2 nm Al_2_O_3_ | W/ | Au |
| --- | --- | --- | --- | --- |
| *b* | Au | 2 nm SiO_2_ | W/ | Au |
| *c* | Ti/Au | 2 nm SiO_2_ | W/ | Au |


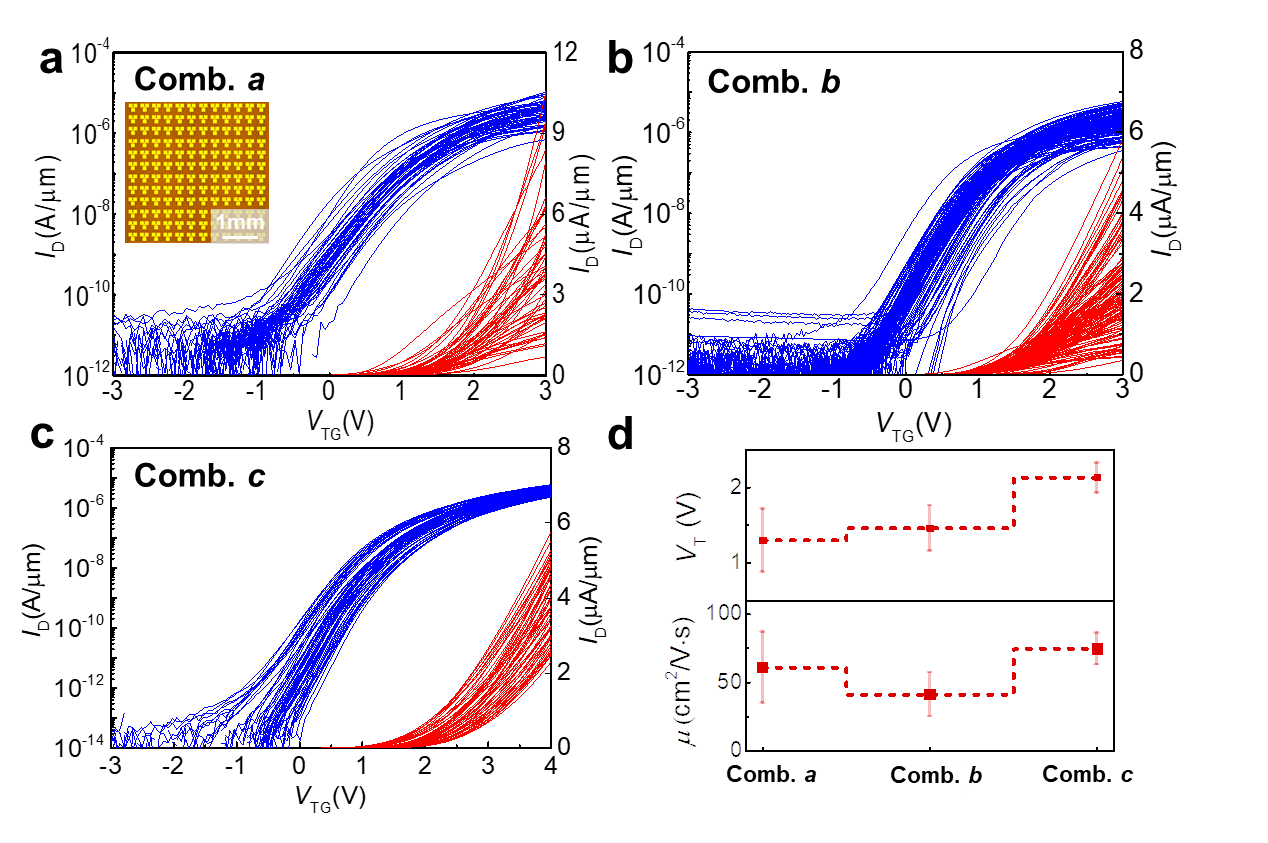


**Supplementary Figure 9.** Electrical performance for top-gated CVD MoS_2_ FETs array in different batches. **a-c,** *I*_D_-*V*_TG_ curves of top-gated CVD MoS_2_ FETs array on wafer-scale substrates in different batches (a-c in Supplementary Table 7) at *V*_DS_ = 0.5 V. **d,** The extracted *V*_T_ and mobility with error bars for devices prepared through recipes a-c. The error bars indicate standard error.

The results show that MoS_2_ TG-FETs fabricated by combination *c* exhibit a more positive *V*_T_, large *I*_on_/*I*_off_, and higher uniformity, which is the best result for further circuit fabrication.

Here, we provide a complete version of recipe *c*:

| *Contact electrodes*: Patterned by photolithography and deposited using Electronic Beam (E-beam) evaporation. Thickness: 10 nm Ti (sticking layer) and 50 nm Au. Evaporation rate: 0.1 Å/s for Ti and 1.0 Å/s for Au. Before evaporation sample was vacuum annealed in an E-beam evaporator at 150 ^o^C for 1 hour. |
| --- |
| *MoS_2_ channel formation*: CF_4_ plasma etching in an ICP (inductively coupled plasma) facility. After etching, the sample was immediately transferred to the E-beam evaporator and annealed at 150 ^o^C for 1 hour for the subsequent seeding layer deposition. |
| *Seeding layer*: 2-nm-thick SiO_2_ seeding layer was deposited by E-beam evaporation with an evaporation rate <0.1 Å/s. Then the sample was immediately transferred into a tube furnace and annealed in an oxygen atmosphere at 100 ℃. |
| *High-k dielectric layer*: 20-nm-thick HfO_2_ layer grown by atomic layer deposition (ALD) at 180 °C, where [Hf(N(CH_3_)_2_)_4_] and H_2_O are used as the precursors and N_2_ as the carrier gas. After ALD growth, the sample was annealed in an oxygen atmosphere at 150 ℃ for 1 hour. |
| *Top gate:* 40-nm-thick Au by E-beam evaporation with an evaporation rate <0.5 Å/s. After the TG deposition, rapid thermal annealing (RTA) was performed at 150 ℃ for 5 min. |

It is noteworthy that recipe *c* is not simply a combination of all best parameters in each processing step from Sections 4–8, and the evaluation score (Fig. 2e) of recipe *a* and *b* are indeed lower than that of *c*. Thus it confirms the effectiveness of our ML method for fabrication optimization indeed works.

Although ML represents a computer-aided learning process from large amounts of device data, the optimization process guided by ML also reveals some underlying physics that can explain our experimental data. For example, in Supplementary Table 7, recipe c has the highest score ranked by the ML algorithm, in which both contact (Ti/Au) and seeding layer (SiO_2_) are not the best options obtained by single-step optimizations. It can be partially explained by: 1) the addition of Ti as a buffer layer between MoS_2_ film and Au electrodes can effectively increase the adhesion of contacts, and its slightly smaller work function is beneficial to reduce the contact resistance and increase the on-state current. 2) A 2-nm-thick SiO_2_ can reduce the damage to MoS_2_ by the growth of HfO_2,_ but its defect level is also high due to the physical vapor deposition method. However, the subsequent annealing can likely repair the oxygen defects in the SiO_2_ layer, thus substantially improving the quality of the dielectric layer and the transistor’s electrostatic control capability.

**Supplementary Note 10 - Low-frequency 1/f noise in top-gated MoS_2_ FETs**

Low-frequency noise measurements were gathered with a semiconductor parameter analyzer (Agilent 4156C), current amplifier (Stanford SR570), and spectrum analyzer (Agilent 35670A) in a manual probe station (Cascade). A schematic diagram of the measurement setup is shown in Supplementary Figure 10.


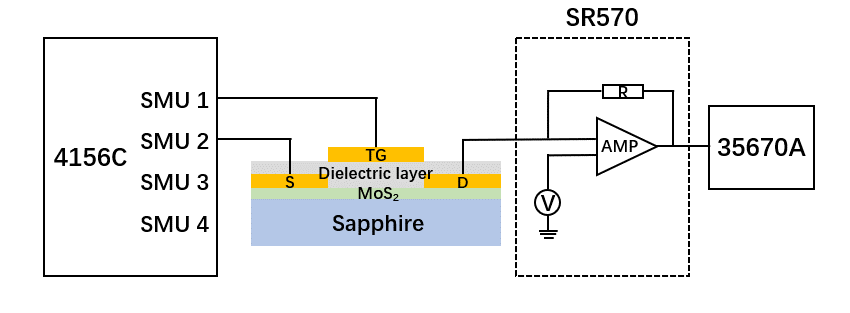


**Supplementary Figure 10.** Schematic diagram of reliability testing equipment of top-gated MoS_2_ FET.

In a working FET, the current fluctuates within a small range due to electrical noise. It is generally represented by power spectral density, i.e., noise power per unit frequency.

Border traps can exchange charge with the channel through carrier capture or emission by tunneling when the gate voltage is swept. The border trap density can be further extracted from the low-frequency noise in the transistor, as shown in Supplementary Figure 11. Here, the noise power spectra normalized against the drain current *I*_D_ are extracted, as shown in Supplementary Figure 11a.


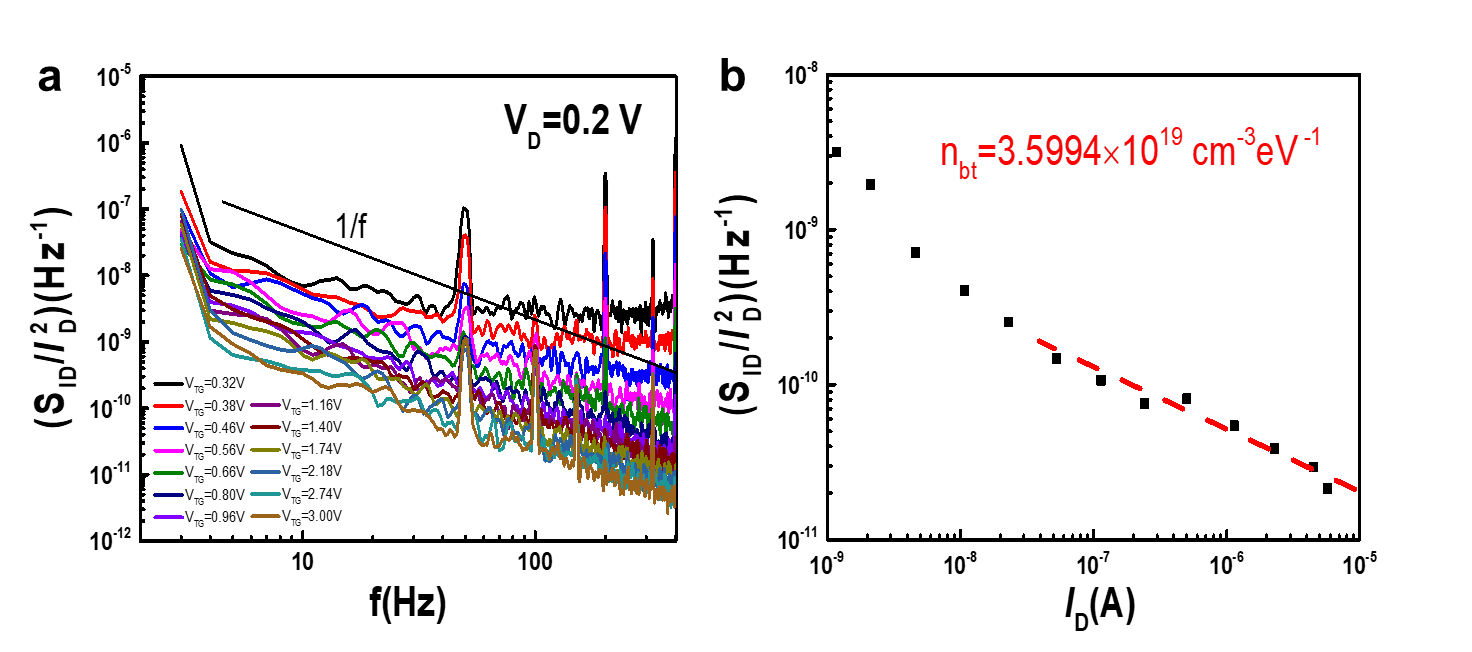


**Supplementary Figure 11.** Low frequency 1/f noise characteristics. **a,** Normalized noise power spectra (𝑆_𝐼𝐷_/I_D_^2^) as a function of *f*. **b,** 𝑆_𝐼𝐷_/*I*_D_^2^ as a function of *I*_D_ at 100 Hz. The dashed line shows a corresponding linear fit.

In order to analyze the border trap intensity, the values of S_ID_/*I*_D_^2^ at 100 Hz are plotted as a function of *I*_D_ in Supplementary Figure 11b. The trap density N_bt_ can be extracted from the following equation:

$\frac{s_{I_{d}}}{I_{d}^{2}}=\frac{kT}{\gamma fWL}{(\frac{1}{N}+\alpha\mu)}^{2}N_{bt}$  (6)

where *W* is the channel width, *L* is the channel length, *μ* is the mobility, *T* is temperature, *k* is Boltzmann’s constant, *f* = 100 Hz, the tunneling coefficient *γ* is typically taken to be 10^8^ cm^−1^, the areal density of the carriers is $qN=C_{OX}(V_{GS}-V_{th})=\frac{I_{D}}{(\frac{W}{L})\mu V_{DS}}$, and α is the scattering coefficient.

By fitting discrete points in Supplementary Figure 11b, the *N*_bt_ values in the accumulation regime were extracted using MATLAB and found to be 3.5994×10^19^ cm^−3^ eV^−1^, which is lower than the value stated in previous reports ^6-8^.

**Supplementary Note 11 - Analysis of inverters with different pull-up transistors**

For circuit-level application, we need to increase the noise margin of the inverter. Based on the level-62 RPI model a simulation result is shown in Supplementary Figure 12a, where different *V*_T_ values are set for load transistor M1 to increases the driving current when *V*_G_ = 0 V. Guided by the simulation results, metals with different work functions were used to prepare TG for pull-up transistor (M1) in the inverter circuit, Experimental results are shown in Supplementary Figure 12b, when Al is used as the top gate metal, the switching point of *V*_TC_ is 1.5 V and a largest noise margin can be obtained.


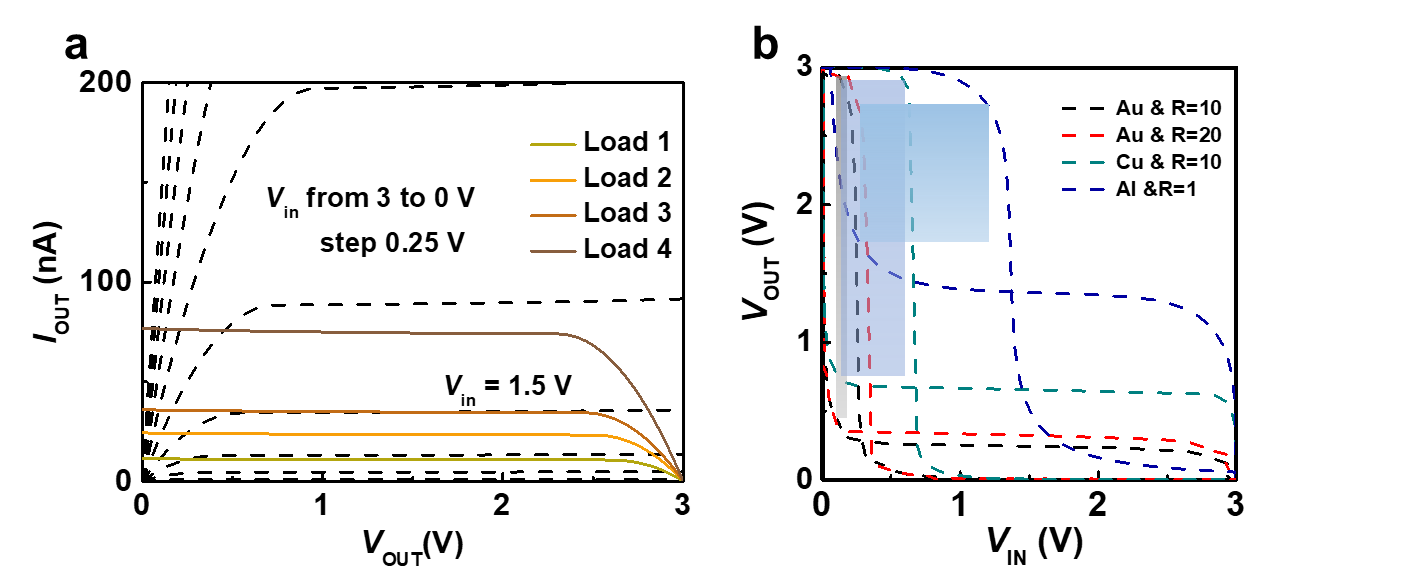


**Supplementary Figure 12.** Performance analysis of the pseudo-NMOS inverters. **a,** Simulation results of output characteristics (*I*_OUT_ vs. *V*_OUT_) for pull-down transistor (M2) when *V*_IN_ is scanned from 0 V to 3 V in 0.25 V increments. The solid lines are the output characteristics of pull-up transistors with different *V*_T_. **b,** Experimental voltage transfer characteristics with pull-up transistor designed by different TG metals and transistor geometry *R*=(W/L)_M1_/(W/L)_M2_.

**Supplementary Note 12 - Analog amplifier composed of an inverter based on MoS_2_ FET**

Thanks to its high voltage gain (>20), the MoS_2_ inverter is a promising analog amplifier for small AC signals. By applying an AC signal with a DC bias on the gate electrode of the driver FET, an amplified voltage can be obtained at the output port. To reach maximum output voltage swing, the DC bias value is carefully controlled to set the inverter in the middle of the steepest region of the voltage transfer curve. We achieved this by applying a 920 mV DC bias and mixing 20 mV AC signals on the gate bias. The frequency of the AC signal was set to be 0.1 Hz due to the limited sample rate of the measurement equipment. The input and output signals are displayed in Supplementary Figure 13, where the output voltage (*V*out-amp) has a phase difference of 180^o^ with respect to the input voltage (*V*in-amp). It is shown that the gain *V*_in-amp_/*V*_out-amp_ is larger than 100, indicating the amplifier circuit has desired functionality. The frequency range can be further extended by reducing the parasitic capacitance in the overlap area and increasing the transistor’s transconductance.


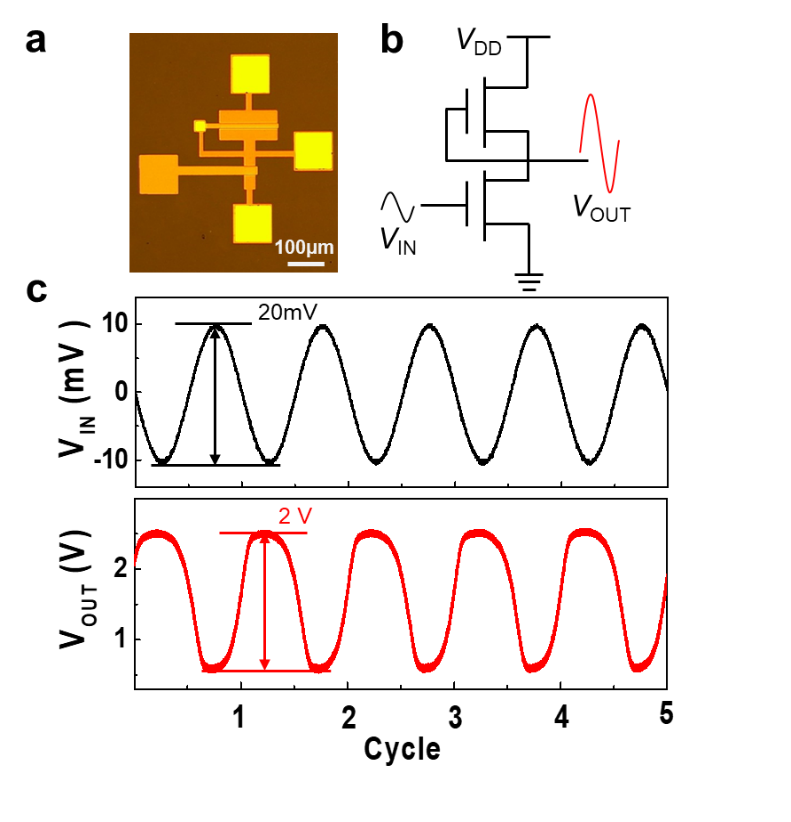


**Supplementary Figure 13.** Small signal amplification of the pseudo-NMOS inverter. **a,** Microscope image and **b,** schematic diagram of the inverter built from a pseudo n-type circuit. **c,** Measured waveforms from the inverter with 20 mV input and ~2 V output indicate high gain (>100).

**Supplementary Note 13 - Measurement results of various basic logic circuit units**

In addition to the negative edge-triggered D flip-flop and 1-bit full-adder mentioned in the main text, we also prepared and tested a 1-stage inverter, 2-stage inverter, NAND, NOR, XNOR, XOR, LATCH, and 2-bit adder, as shown in Supplementary Figure 14 and 15.


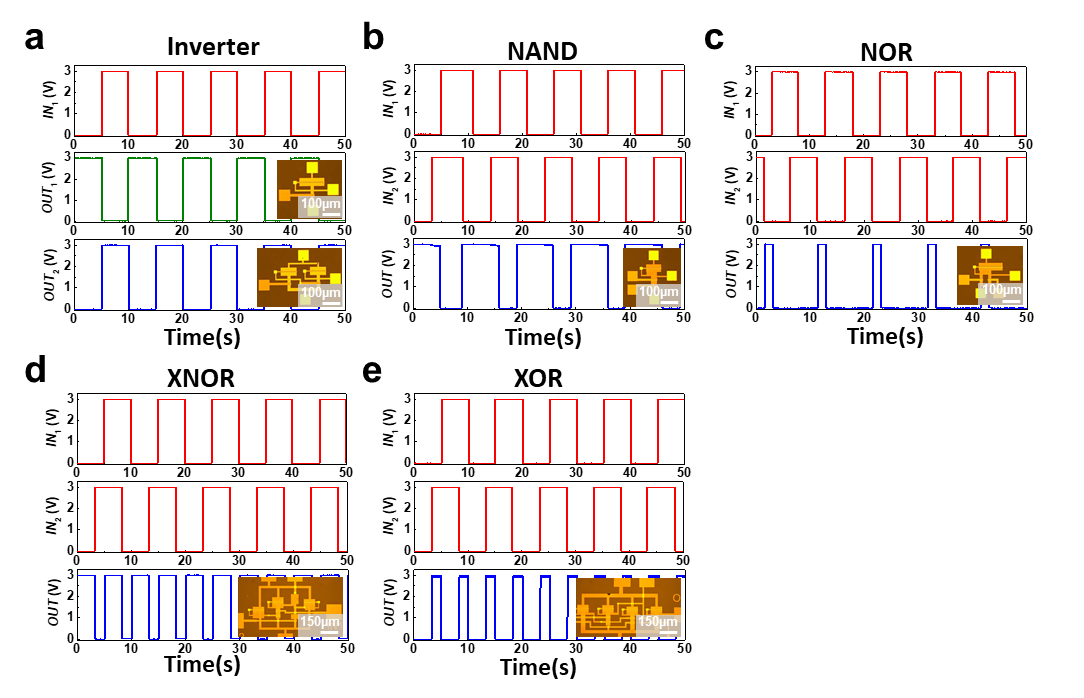


**Supplementary Figure 14.** Electrical measurement of basic logic circuits. Micrograph and measurements from the fabricated **a,** inverter, **b,** NAND, **c,** NOR, **d,** XNOR, and **e,** XOR.


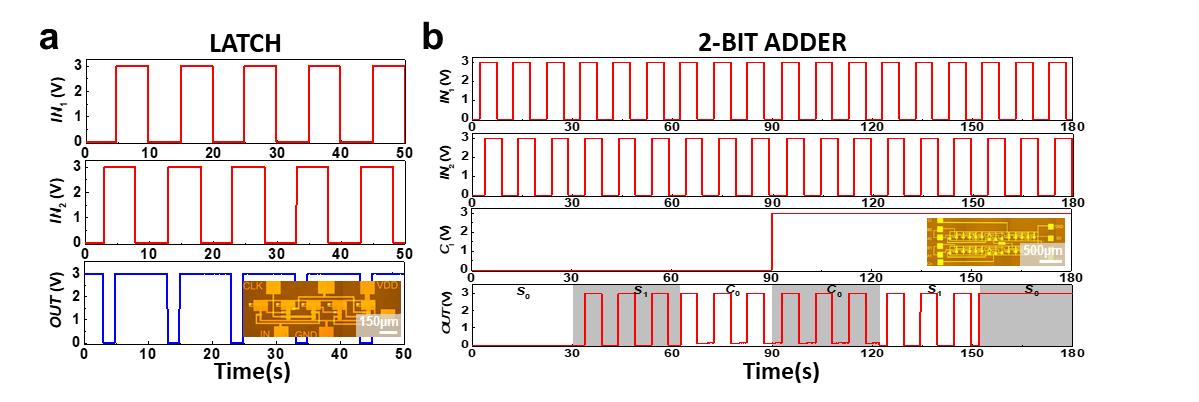


**Supplementary Figure 15.** Electrical measurement of basic logic circuits. Micrograph and measurement results from the fabricated **a,** LATCH and **b,** 2-bit adder.

**Supplementary Note 14 - The limiting factor for high frequency operation of MoS_2_ integrated circuits**

RC delay is the main limiting factor for the high operating frequency of the circuit, which is correlated with the load capacitance and resistance in the circuit, as well as the gate capacitance, parasitic capacitance and equivalent resistance of the transistors.

To simplify the situation, we exclude the influence of additional load capacitance and resistance in the circuit, so the operating speed mainly depends on the response speed of the MoS_2_ FETs, which is determined by the cut-off frequency $f_{T}=\frac{g_{m}}{2\pi C_{G}}$, where $g_{m}$ is the transconductance of the channel and $C_{G}$ is the equivalent gate capacitance.

In Supplementary Figure 16, we measured the experimental gate capacitance $C_{G}$, which is approximately 4.5 pF when $V_{g}\epsilon\left[ 1.5,3.0 \right] V$, and the transconductance $g_{m}\approx3.8 \mu S$ when $V_{g}\epsilon\left[ 1.5,3.0 \right] V$. Therefore, the maximum value of $f_{T}$ is approximately 134.5 kHz, which can be treated as a reference value of the possible circuit operating frequencies.


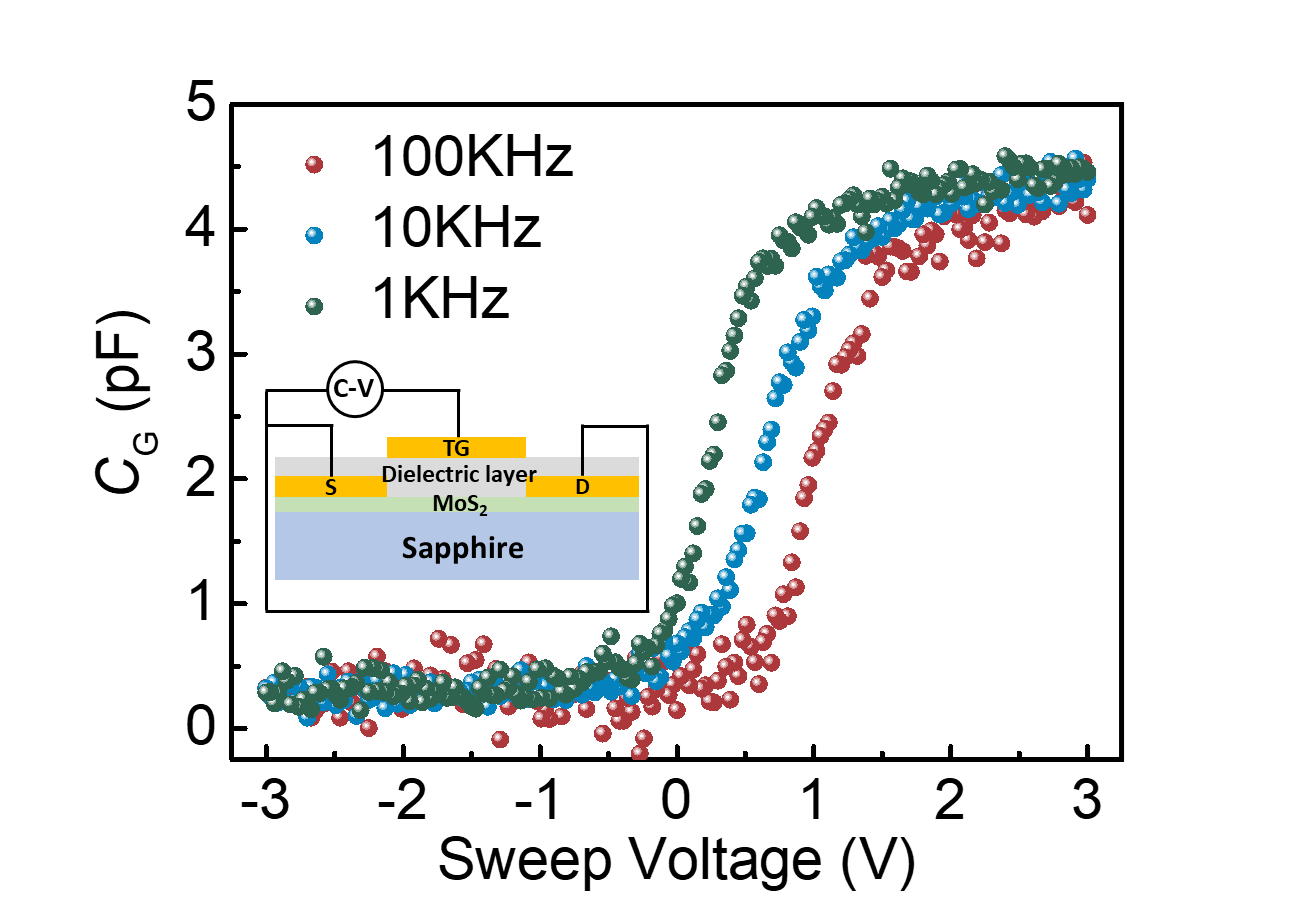


**Supplementary Figure 16.** Capacitance-voltage measurement of a MoS_2_ TG-FET under different frequencies (1 kHz, 10 kHz, and 100 kHz), the insert is a measurement schematic diagram.

**Supplementary Note 15 - Schematic diagram of DRAM testing setup**

DRAM testing: A waveform generator was used to generate signals applied to the gate and drain bias of the DRAM transistor, as shown in Supplementary Figure 17a. The source was collected with an equivalent circuit, as shown in Supplementary Figure 17b.


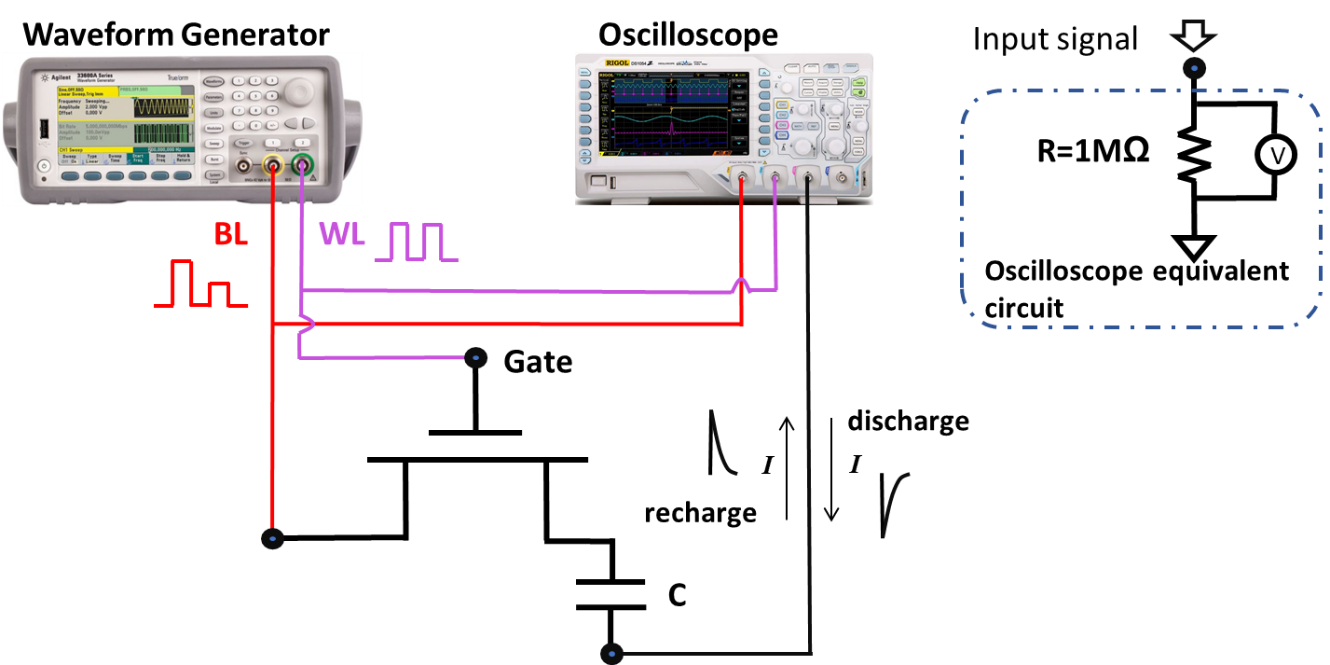


**Supplementary Figure 17.** Schematic diagram of DRAM testing equipment and equivalent oscilloscope circuit.

The characteristics of reading charge pulse after different hold time ranging from 10 to 8000 ms at the read operation are shown in Supplementary Figure 18. The reading charge pulse changes from negative to positive as the holding time increases, which means that the charge of DRAM is discharging with the hold time increasing.


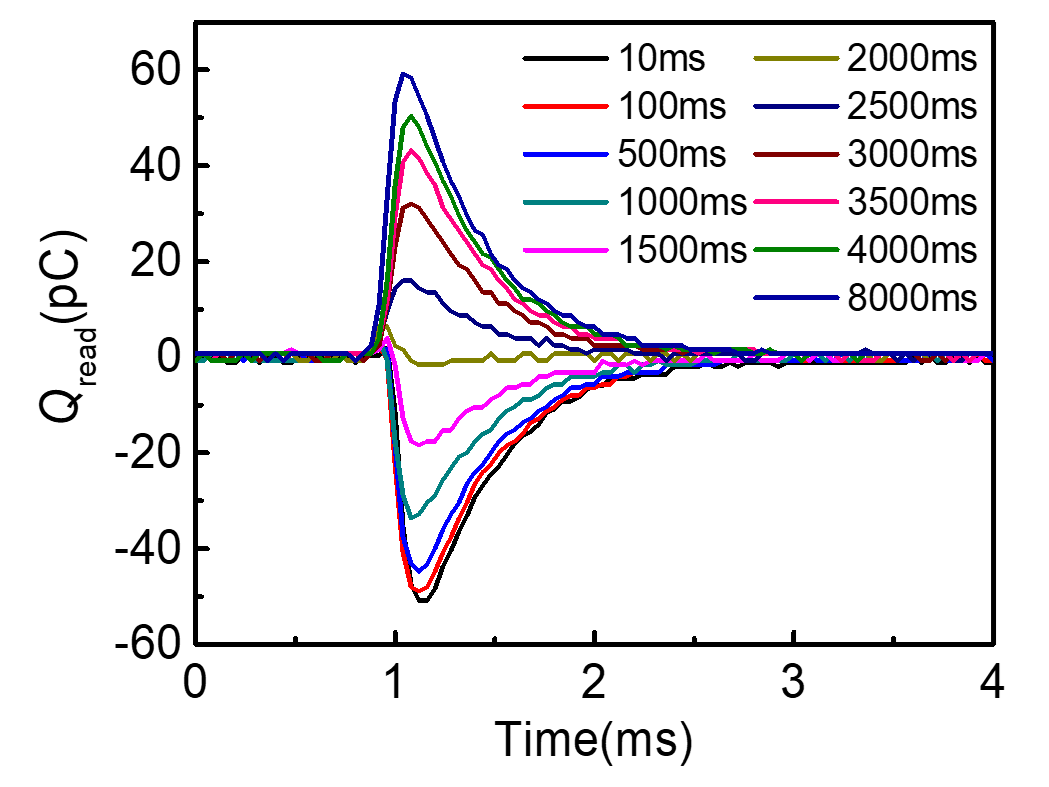


**Supplementary Figure 18.** The characteristics of reading charge pulse after different hold time at the read operation.

**Supplementary Note 16 - Photoelectric characteristics for top-gated MoS_2_ FETs**

In order to characterize the photoelectric characteristics of the top-gated MoS_2_ FETs, we measured the transfer characteristics while the devices were illuminated with different wavelengths and power density values at *V*_DS_ = 1 V. Further, the photocurrent can be extracted, as shown in Supplementary Figure 19. The responsivity spectra at different power density values were also calculated.

The time-resolved photoresponse in the top-gated MoS_2_ phototransistor is shown in Supplementary Figure 20, where the data were gathered at *V*_TG_ = −1 V and *V*_DS_ = 1 V while illuminated with 1.55 mW·cm^−2^ at 550 nm. A rise time of 1.2 s and fall time of 5.76 s were observed in the photocurrent measurements.

Two mechanisms can influence the photoconductivity of a transistor: the photovoltaic (PV) and the photoconductivity (PC) effects^9^. The photovoltaic effect is described as a shift in transistor *V*_T_ due to charges transfer from the channel to the MoS_2_/dielectric interface or nearby molecules, making the photodetectors respond slowly. The photoconductive effect is caused by the capture of carriers in band tail states in MoS_2_ itself, and the response speed is relatively high.

For the photodetector in this work, the dominant mode of operation should be the photogating effect^9,10^. Under illumination, free electron-hole pairs are generated. The trap states are occupied by photo-generated holes and act as a localized floating gate strongly modulating the channel conductance. Therefore, the transfer curves under illumination are horizontally left shifted from that of the dark state. Due to the slow detrapping process, the long lifetime of the photogenerated carriers results in high gain but slow response speed. The response speed of the MoS_2_ photodetector can be further improved by modifying the device structure and operating mode.


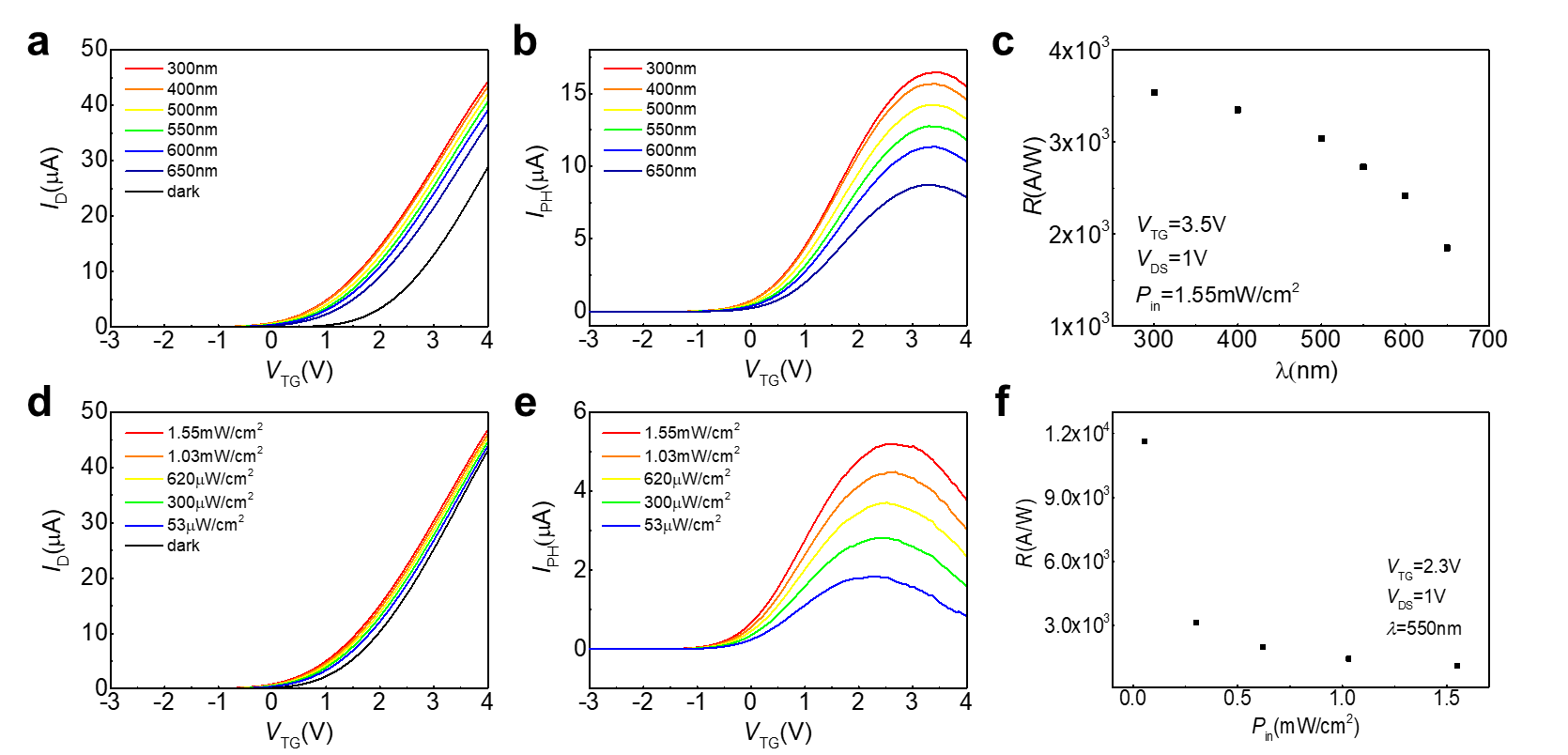


**Supplementary Figure 19.** The photoelectric properties of a phototransistor. *I*_D_-*V*_TG_ curves for a TG MoS_2_ FET in darkness and while illuminated with **a,** different wavelengths and **d,** different *P*_in_ values at *V*_DS_ = 1 V. *I*_PH_-*V*_TG_ curves for a top-gated MoS_2_ FET illuminated with **b,** different wavelengths and **e,** different *P* in values. **c,** Corresponding wavelength and **f,** *P*_in_ dependence of *R*.


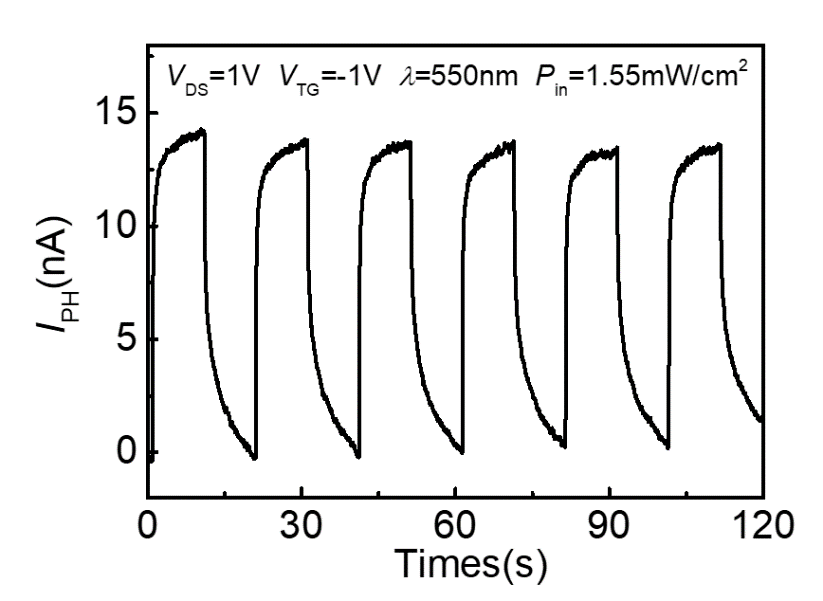


**Supplementary Figure 20.** Time-resolved photoresponse of the TG MoS_2_ phototransistor with *V*_TG_ = −1 V and *V*_DS_ = 1 V under illumination with 1.55 mW·cm^−2^ at 550 nm.

Detailed photocurrent characteristics from 9×9 TG MoS_2_ FET arrays mentioned in Fig. 4i are shown in Supplementary Figure 21 while illuminated with white light at *V*_DS_ = 0.5 V.


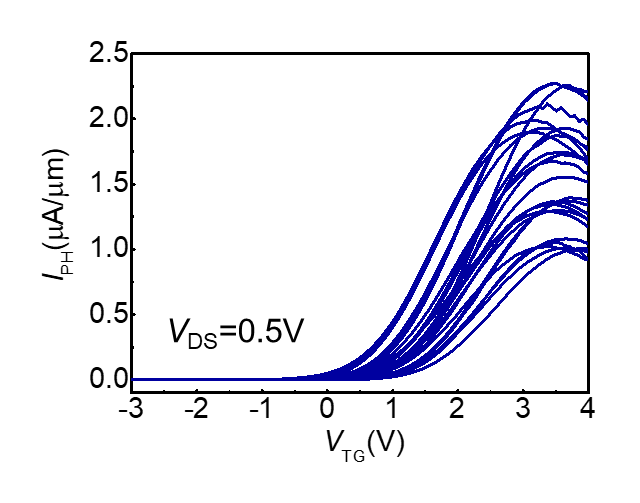


**Supplementary Figure 21.** Photocurrent characteristics of 9×9 top-gated MoS_2_ FET arrays shown in Fig. 4i.

**Supplementary Note 17 - Discussion of the origins for failed circuits in Fig. 5.**

We attribute that the relatively low wafer-scale yield to three main issues:

1) The quality and uniformity of MoS_2_ films, such as grain boundaries and local defects, are detrimental to the yield of wafer-scale integrated circuits. It can be improved by a further upgrade of synthesis methods and facilities.

2) As shown in Supplementary Figure 9, the uniformity of MoS_2_ FETs still depends on the processing recipes. The transistor size might also influence the yield since the length and width of the MoS_2_ FETs are not variable parameters in our ML algorithm.

3)The quality of processing tools and cleanroom grade (10^4^) is relatively low comparing with the industrial standard. A higher standard laboratory is necessary for future serious MoS_2_ IC fabrication.

**Supplementary Note 18 - Comparison of our work with recently published results**

Supplementary Table 8 summarizes the performance of large-scale MoS_2_ FETs from previously published results and our work. Among these results, our MoS_2_ TG-FETs exhibit a satisfactory comprehensive performance, and our work presents the maximum transistor number in a functional circuit.

**Supplementary Table 8.** Comparison of MoS_2_ FET performance with recently published results

| Area | *V*_DS_ (V) | *I*_on_ (A) | *I*_off_ (A) | Max  *I*_on_/*I*_off_ | Max. μ  (cm^2^/Vs) | *V*_T_ (V) | Max.FET # in a working IC | W/L | Gate Structure | Ref |
| --- | --- | --- | --- | --- | --- | --- | --- | --- | --- | --- |
| ~50mm^2^ | 5 | 9×10^-5^ | 10^-12^ | 10^8^ | ~3 | ~0.65 | 115 | 45/2 | BG | ^11^ |
| 2mm×3mm | 2 | 2×10^-5^ | 10^-11^ | 10^6^ | 3 | ~1.3 | 3 | 45/3 | TG | ^12^ |
| 4 inch | 3 | 10^-3^ | 10^-13^ | 10^10^ | ~55 | ~1.7 | 12 | 30/6 | BG | ^13^ |
| - | 3 | 10^-3^ | 10^-13^ | 10^10^ | ~50 | 0.54 | 9 | 30/4 | BG | ^14^ |
| 1cm×0.5cm | 1 | ~10^-5^ | 10^-14^ | 10^8^ | ＞40 | -2 | 3 | 1/1 | TG | ^15^ |
| - | 1.5 | 10^-5^ | 10^-14^ | 10^9^ | 80 | 2.41 | 10 | 30/4 | BG | ^16^ |
| 5mm×5mm | 8 | 12×10^-5^ | 10^-14^ | 10^10^ | ~20 | ~3.2 | 12 | 4 | BG | ^17^ |
| 2 inch | 0.5 | 5.65×10^-5^ | 10^-14^ | 10^9^ | ~88 | 2.47 | 156 | 30/20 | TG | **This work** |

**Supplementary References**

1 Svetnik, V. *et al.* Random Forest: A Classification and Regression Tool for Compound Classification and QSAR Modeling. *Journal of Chemical Information and Computer Sciences* **43**, 1947-1958 (2003).

2 Archer, K. J. & Kimes, R. V. Empirical characterization of random forest variable importance measures. *Computational Stats & Data Analysis* **52**, 2249-2260 (2008).

3 Wang, H. *et al.* Integrated Circuits Based on Bilayer MoS_2_ Transistors. *Nano Letters* **12**, 4674-4680 (2012).

4 Ayers, J. E. Digital integrated circuits: analysis and design; CRC Press: Boca Raton, FL. (2004).

5 Kaushik, N. *et al.* Reversible hysteresis inversion in MoS_2_ field effect transistors. *npj 2D Materials and Applications* **1**, 34 (2017).

6 Srinivasan, P., Olubuyide, O., Choi, Y. S. & Marshall, A. in *International Electron Devices Meeting.* 27.24.21-27.24.24. DOI:10.1109/IEDM.2010.5703433

7 Sangwan, V. K. *et al.* Low-Frequency Electronic Noise in Single-Layer MoS_2_ Transistors. *Nano Letters* **13**, 4351-4355 (2013).

8 Xie, X. *et al.* Low-Frequency Noise in Bilayer MoS_2_ Transistor. *ACS Nano* **8**, 5633-5640 (2014).

9 Furchi, M. M., Polyushkin, D. K., Pospischil, A. & Mueller, T. Mechanisms of Photoconductivity in Atomically Thin MoS_2_. *Nano Letters* **14**, 6165-6170 (2014).

10 Fang, H. & Hu, W. Photogating in Low Dimensional Photodetectors. *Advanced Science* **4**, 1700323 (2017).

11 Wachter, S., Polyushkin, D. K., Bethge, O. & Mueller, T. A microprocessor based on a two-dimensional semiconductor. *Nature Communications* **8**, 14948 (2017).

12 Wang, L. *et al.* Electronic Devices and Circuits Based on Wafer-Scale Polycrystalline Monolayer MoS_2_ by Chemical Vapor Deposition. *Advanced Electronic Materials* **5**, 1900393 (2019).

13 Li, N. *et al.* Large-scale flexible and transparent electronics based on monolayer molybdenum disulfide field-effect transistors. *Nature Electronics* (2020).

14 Yu, L. *et al.* in *International Electron Devices Meeting.* 32.33.31-32.33.34. DOI: 10.1109/IEDM.2015.7409814

15 Wang, H. *et al.* in *International Electron Devices Meeting.* 4.6.1-4.6.4. DOI: 10.1109/IEDM.2012.6478980.

16 Yu, L. *et al.* Design, Modeling, and Fabrication of Chemical Vapor Deposition Grown MoS_2_ Circuits with E-Mode FETs for Large-Area Electronics. *Nano Letters* **16**, 6349-6356 (2016).

17 Polyushkin, D. K. *et al.* Analogue two-dimensional semiconductor electronics. *Nature Electronics* **3**, 486-491 (2020).
